# Efficacy and safety of autologous adipose-derived stem cells in subjects with moderate to severe atopic dermatitis: a multicenter, randomized, single-blind, placebo-controlled, phase 2 trial

**DOI:** 10.1186/s13287-025-04763-y

**Published:** 2025-12-02

**Authors:** Joon Seok, Su-Young Kim, Woo Geon Lee, Min Kyung Shin, Dong Hun Lee, Young-Joon Seo, Soyun Cho, Kui Young Park, Sang Wook Son, Sung-Hoon Lee, Jun-Seok Seo, Seong Jun Seo

**Affiliations:** 1 Department of Dermatology, Chung-Ang University Hospital, Chung-Ang University College of Medicine, Seoul, Republic of Korea; 2 Department of Medicine, Graduate School, Chung-Ang University, Seoul, Republic of Korea; 3 Department of Dermatology, Kyung Hee University Hospital, College of Medicine, Kyung Hee University, Seoul, Republic of Korea; 4 Department of Dermatology, Seoul National University Hospital, Seoul National University College of Medicine, Seoul, Republic of Korea; 5 Department of Dermatology, Chungnam National University Hospital, Daejeon, Republic of Korea; 6 Department of Dermatology, Seoul Metropolitan Government-Seoul National University Boramae Medical Center, Seoul, Republic of Korea; 7 Department of Dermatology, Korea University Ansan Hospital, Korea University College of Medicine, Ansan, Republic of Korea; 8 Institute of Cell Biology and Regenerative Medicine, EHLBio Co., Ltd, Uiwang-si, Republic of Korea; 9 Department of Dermatology, Soonchunhyang University Cheonan Hospital, Cheonan, Republic of Korea

**Keywords:** Atopic dermatitis, Autologous adipose-derived stem cells, Mesenchymal stem cells

## Abstract

**Background:**

Atopic dermatitis (AD) is a chronic skin condition affecting patients’ well-being, but conventional treatments have limitations. Mesenchymal stem cells (MSCs) present a promising option for AD therapy, though large-scale clinical studies are scarce. This study aimed to assess the efficacy and safety of autologous adipose tissue-derived MSC (AtMSC) in moderate to severe AD refractory to conventional treatments.

**Methods:**

This multicenter, randomized, single-blind, placebo-controlled, phase 2 trial included 114 participants. Participants received two intravenous injections of AtMSCs or placebo at 4-week intervals. Clinical assessments, comprising Eczema Area and Severity Index (EASI), Scoring Atopic Dermatitis (SCORAD), and Investigator’s Global Assessment (IGA), were performed every 4 weeks for 16 weeks total. Biomarker analyses were conducted using ELISA.

**Results:**

Statistically significant differences between the treatment and placebo groups in EASI total score were observed at 8, 12, and 16 weeks (*P* = .0.017, 0.015, < 0.001). At week 16, 23.7% [14/59] of participants in the treatment group achieved a 75% or greater reduction in EASI total score (EASI-75), compared to 7.3% [4/55] in the placebo group, with a statistically significant difference (*P* = .016). In addition, SCORAD, disease severity, and IGA score were also improved in the treatment group compared to the placebo group. Furthermore, the change in TARC levels from baseline to week 16 was significantly different between the treatment and placebo groups.

**Conclusions:**

AtMSC therapy improved moderate to severe AD, offering a promising treatment option with potential applications in chronic inflammatory diseases. Further investigation, including double-blind phase 3 trials, is needed to confirm these findings and explore additional biomarkers.

*Trial registration:* ClinicalTrials.gov, Identifier: NCT04137562; October 21, 2019; https://clinicaltrials.gov/study/NCT04137562.

**Supplementary Information:**

The online version contains supplementary material available at 10.1186/s13287-025-04763-y.

## Introduction

 Atopic dermatitis (AD) is a recurrent, chronic inflammatory skin condition characterized by intense itching and a spectrum of phenotypes [1]. Studies have shown that moderate to severe AD significantly affects a patient’s emotional and psychological well-being, beyond the cutaneous symptoms [2, 3]. AD has a lifetime prevalence of 10–20% in children and 3–7% in adults, presenting a considerable burden to public health worldwide [1, 4, 5].

The severity of AD dictates the treatment approach, with severe cases often requiring systemic immunosuppressants such as steroids, cyclosporine, and JAK inhibitors [6, 7]. However, prolonged use of immunosuppressants may lead to serious side effects and toxicity. Patients with moderate to severe AD can benefit from biologics targeting cytokines such as IL-4/13, IL-13, IL-31, yet many patients do not achieve complete or near-complete remission of their condition [8–10].

Mesenchymal stem cells (MSCs), multipotent stem cells obtainable from various tissues including umbilical cord, bone marrow, and adipose tissue, are distinguished by cell-surface markers such as CD73, CD90, and CD105, and the absence of CD45, CD34, CD14, CD19, CD11b, and HLA-DR [11]. MSCs are pivotal in anti-inflammatory, tissue repair, and antitumorigenic processes [12]. They exert a suppressive effect on the activation of immune cells such as T cells, B cells, dendritic cells, and natural killer cells, through interactions with the innate and adaptive immune systems [13–15]. MSCs have demonstrated promise in improving allergic conditions such as AD and asthma in mouse models [16, 17]. Their unique characteristics make MSCs an attractive option for treating allergic diseases.

The impact of MSC therapy on AD has been the subject of several studies in clinical trials [18, 19]. Nevertheless, the effectiveness and safety of MSC therapy in large-scale clinical evaluations for AD remain inadequately understood. In this context, we conducted a clinical trial to assess the efficacy and safety of autologous adipose tissue-derived MSC (AtMSC) therapy in 114 adult patients with moderate to severe AD who were unresponsive to conventional treatments. Furthermore, we measured cytokines in the blood of participants to provide further insights.

## Methods

### Study design and subjects

Based on Hanifin and Rajka’s criteria, 118 participants were selected following the inclusion and exclusion criteria. A total of 130 participants underwent screening at six clinical trial sites after providing written informed consent. Out of these, 118 were randomized (treatment group: 60, placebo group: 58). Among the randomized participants, 114 (treatment group: 59, placebo group: 55) received the study medication. However, 11 participants (treatment group: 6, placebo group: 5) discontinued, leaving 107 (treatment group: 54, placebo group: 53) who completed the study (refer to Supplementary Table 1). We enrolled participants experiencing frequent AD symptom recurrences not sufficiently managed with topical corticosteroids or systemic immunosuppressants. The main inclusion criteria were moderate to severe AD (SCORAD > 20), ages between 19 and 70 years, and persistent symptoms for at least six months. Concurrent medications included antihistamines and topical steroid ointment (grade 6–7), along with emollients during AtMSC treatment. Participants were randomly allocated in a 1:1 ratio to receive AtMSC treatment using stratified block randomization, with the stratification factor being study site and a block size of 4 or 6. Each participant received two doses of either intravenous AtMSC solution or placebo (0.9% normal saline) at four-week intervals. Safety and efficacy were evaluated at four-week intervals over 16 weeks following the initial dose of the study medication.

### Isolation and culture of human adipose tissue-derived mesenchymal stem cells

AtMSCs were isolated from human adipose tissue obtained via liposuction [20]. The adipose tissue was washed with α-minimum essential medium (α-MEM; Gibco, NY, United States) and digested with a 0.1% collagenase (type I, Gibco, NY, United States) solution at 37 °C for 30 min. The cell pellet, obtained by centrifugation at 1500 rpm for 5 min, was resuspended in α-MEM containing 8% fetal bovine serum (Gibco, Mulgrave Victoria, Australia) and filtered through a 100 μm nylon mesh. The cell suspension was incubated at 37 °C in 5% CO_2_ for two days; unbound cells were removed by washing. Cells were passaged five times by cell culture, and AtMSCs from the donor were used after confirming the characteristics for MSCs by assessing surface markers (CD73, CD90, CD105; positive markers, CD34, CD45; negative markers) using FACSVerse (BD).

### Procedures

In this study, participants received two IV injections with a dose of 1.0 × 10^8^ cells at four-week intervals, a dose confirmed to be safe and effective in the phase 1 clinical trial (data not shown). For the placebo group participants, compensatory treatment was offered to those who desired it. The AtMSC solution was prepared and injected within 24 h of preparation and stored at 2–8 °C. Before injection, 10 mL of AtMSC solution was combined with 320 mL of normal saline. The study medication and placebo differed in formulation and composition; hence, the investigator remained blinded, while participants and independent evaluators were also blinded. To maintain single-blinding in this study, participants were required to wear an eye mask during the administration of the study medication. At each clinical trial site, a physician qualified to assess the study was designated as an independent evaluator, was blinded to the participants’ treatment group, and assessed efficacy at an independent location.

### Clinical outcome assessment

The Eczema Area and Severity Index (EASI, score range from 0 to 72), Scoring Atopic Dermatitis (SCORAD, from 0 to 103), the grading of the severity of AD, and the Investigator’s Global Assessment (IGA, from 0 to 5) (Supplementary Table 2) were measured at baseline and each subsequent visit, with an endpoint at 16 weeks. At every study visit, each scale of AD was assessed using scoring parameters. An additional outcome parameter was the number of participants exhibiting a reduction in EASI or SCORAD score by more than 50% and 75%. Subjective and objective assessments were conducted at every visit by the same investigator. These included a comprehensive physical examination, vital signs, review of concomitant medication, and blood tests. Any adverse effects were documented.

### Biomarkers

Throughout the treatment cycle, blood was drawn. Serum samples were aliquoted, frozen at − 80 °C, and thawed immediately before analysis. ELISA was used to measure Prostaglandin E2 (PGE2) (R&D, USA), Eosinophil Cationic Protein (ECP) (Thermoscientific, Sweden), TGF-β1 (R&D, USA), IL–4, 5, 6, 8, 13 (Millipore, USA), IL-31 (Raybiotech, USA), and thymus and activation regulated chemokine (TARC) (R&D, USA). The assays were conducted according to the manufacturer’s instructions. All measurements were made individually.

### Safety assessment

An adverse event (AE) is any untoward and unintended sign, symptom, or disease occurring in a clinical trial subject who has received an investigational medicinal product (IMP), and does not necessarily have a causal relationship with the IMP. A treatment-emergent adverse event (TEAE) refers to an AE that was not present before IMP administration but occurred afterward, or a pre-existing symptom that worsened following IMP administration. If the relationship between the AE and the IMP is assessed as “related”, the event is classified as an adverse drug reaction (ADR).

### Statistical analysis

For analysis, the obtained data were categorized into the Full Analysis Set (FAS), Per Protocol Set (PPS), and Safety Set (SS). The primary analysis population was defined as the FAS, which included all randomized participants who received at least one dose of the study medication and had at least one efficacy assessment. Efficacy endpoints were analyzed using both FAS and PPS, while safety endpoints were analyzed using the Safety Set (SS, all participants who received at least one dose of the study medication and underwent at least one safety assessment). Changes in EASI, SCORAD 50/75, and IGA from baseline, as well as the proportion of participants requiring rescue medication during the study, were evaluated for between-group differences using Chi-square or Fisher’s exact test (SAS 9.4; SAS Institute, Cary, NC). Differences in total scores for EASI and SCORAD, as well as changes in biomarkers, were compared between groups using two-sample *t*-tests and Wilcoxon rank sum tests. Within-group changes were analyzed using paired *t*-tests or Wilcoxon signed rank tests. A *P* < .05 was regarded as indicative of statistical significance.

## Results

### Subject subjects

**Fig. 1:**
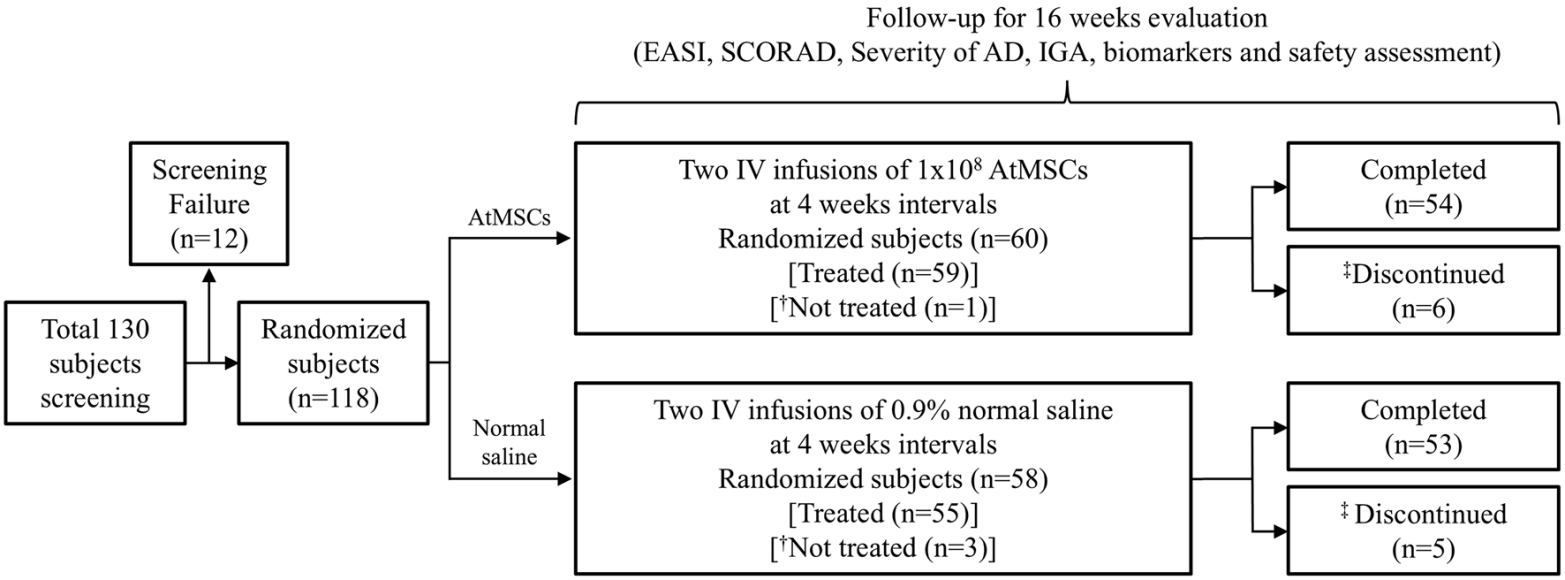
Summary of the disposition of study subjects in clinical trial.* AtMSC* Autologous adipose tissue-derived MSC,* EASI* Eczema area and severity index,* SCORAD* Scoring atopic dermatitis,* IGA* Investigator’s global assessment,* AD* Atopic dermatitis,* IV* Intravenous. ^†^’Not treated’ refers to the group that did not receive infusions of either AtMSC or normal saline. ^‡^’Discontinued’ refers to the combined group consisting of those who were in the ‘Not treated’ group and those who discontinued AtMSC or normal saline infusions

Figure 1 present a summary of the disposition of study subjects in clinical trial. The mean age (standard deviation (SD)) of the participants was 31.21 (10.74) years, with 82 (71.9%) males and 32 (28.1%) females. The mean height (SD) and mean weight (SD) were 169.18 (8.34) cm and 72.43 (15.00) kg, respectively. Participants’ demographics and baseline characteristics showed no statistically significant differences between treatment groups (Table 1). Comorbidities included ‘Mite allergy’ in 35 (30.7%, 104) [treatment group: 20 (33.9%, 62), placebo group: 15 (27.3%, 42)], ‘Allergy to animals’ in 20 (17.5%, 31 cases) [treatment group: 13 (22.0%, 20 cases), placebo group: 7 (12.7%, 11 cases)], and ‘Allergic rhinitis’ in 16 (14.0%, 16 cases) [treatment group: 8 (13.6%, 8 cases), placebo group: 8 (14.6%, 8 cases)], among others (Supplementary Table 3).

**Table 1:** Demographics (full analysis set)

	Treatment group (*N* = 59)	Placebo group (*N* = 55)	Total (*N* = 114)
*Age (years)*			
N	59	55	114
Mean (SD)	30.61(9.93)	31.85(11.61)	31.21(10.74)
Median	28.00	28.00	28.00
Min, Max	19.00, 68.00	19.00, 69.00	19.00, 69.00
*P*-value [1]	0.74[d]		
*Sex*,* n (%)*			
Male	41(69.5)	41(74.6)	82(71.9)
Female	18(30.5)	14(25.5)	32(28.1)
*P*-value [1]	0.55[a]		
*Height (cm)*			
N	59	55	114
Mean (SD)	169.27(8.58)	169.09(8.15)	169.18(8.34)
Median	170.00	169.20	169.75
Min, Max	144.80, 193.60	149.90, 185.20	144.80, 193.60
*P*-value [1]	0.91[c]		
*Weight (kg)*			
n	59	55	114
Mean (SD)	70.81(13.78)	74.17(16.15)	72.43(15.00)
Median	69.90	73.00	71.45
Min, Max	48.60, 117.10	44.30, 136.80	44.30, 136.80
*P*-value [1]	0.18[d]		
*EASI score (baseline)*			
n	59	55	–
Mean (SD)	19.4(7.9)	19.7(9.0)	–
Median	18.8	18.3	–
Min, Max	5.1, 41.1	6.4, 51.8	–
*P*-value [1]	0.90[d]		–
*IGA score (baseline)*			
n	59	55	–
Mean (SD)	3.3(0.8)	3.3(0.7)	–
Median	3.00	3.0	–
Min, Max	2.00, 5.00	2.00, 4.00	–
*P*-value [1]	0.96[d]		–

SD = Standard Deviation, Min = Minimum, Max = Maximum

[1]*P*-value for comparisons between Placebo group and Treatment group: [a] Chi-square test [b] Fisher’s exact test [c] Two-sample *t*-test [d] Wilcoxon’s rank sum test

### Clinical outcomes

**Fig. 2:**
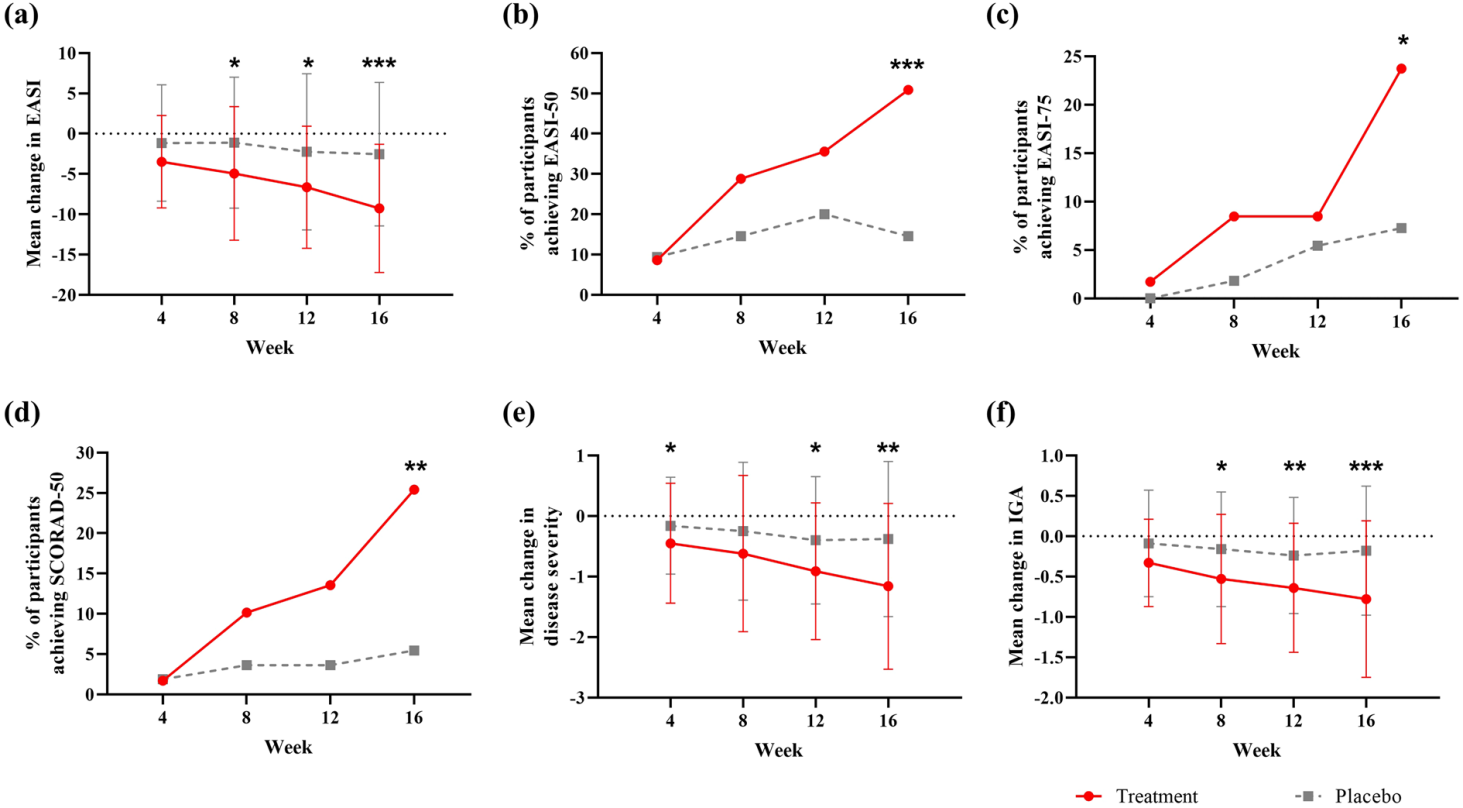
**a** Mean change in EASI total score, **b** Percentage of participants achieving EASI-50, **c** Percentage of participants achieving EASI-75, **d** Percentage of participants achieving a 50% or greater reduction in SCORAD total score (SCORAD-50), **e** Mean change in disease severity and **f** Mean change in IGA score—all measured from baseline at each time point. ^*^P < .05, ^**^P < .01, ^***^P < .001. *P*-values are shown only for statistically significant differences.* EASI* Eczema area and severity index,* SCORAD* Scoring atopic dermatitis,* IGA* Investigator’s global assessment

The mean (SD) EASI total score at baseline showed no statistically significant difference between the treatment and placebo groups. The mean (SD) change in EASI total score from baseline to weeks 4, 8, 12, and 16 was − 3.48 (5.74), − 4.93 (8.28), − 6.63 (7.58), and − 9.26 (7.96) in the treatment group, respectively, and − 1.16 (7.21), − 1.11 (8.12), − 2.25 (9.69), and − 2.54 (8.91) in the placebo group, respectively. Statistically significant differences between the treatment and placebo groups were observed at 8, 12, and 16 weeks (*P* = .017, 0.015, < 0.001) (Fig. 2^2^). The percentage of participants achieving a 50% or greater reduction in EASI total score (EASI-50) from baseline to weeks 4, 8, and 12 was 8.6% [5/58], 28.8% [17/59], and 35.6% [21/59] in the treatment group, respectively, and 9.4% [5/53], 14.6% [8/55], and 20.0% [11/55] in the placebo group, respectively, with no statistically significant difference noted at any of these time points. Nonetheless, the percentage of participants achieving EASI-50 from baseline to week 16 was notably higher at 50.9% [30/59] in the treatment group compared to 14.6% [8/55] in the placebo group, with this difference being statistically significant (*P* < .001) (Fig. 2b). The difference between the treatment and placebo groups (placebo − treatment) was − 36.30% [95% CI: −52.10, − 20.51], with the upper bound of the confidence interval below 0, demonstrating the superiority of the treatment group over placebo. The percentage of participants with a 75% or greater reduction in EASI total score (EASI-75) at weeks 4, 8, 12, and 16 was 1.7% [1/58], 8.5% [5/59], 8.5% [5/59], and 23.7% [14/59] in the treatment group, respectively, and 0.0% [0/53], 1.8% [1/55], 5.5% [3/55], and 7.3% [4/55] in the placebo group, respectively. A statistically significant difference between the treatment and placebo groups was observed at week 16 (*P* = .016) (Fig. 2c).

The SCORAD score also improved over time, with a similar tendency to the change in EASI score (Fig. 2d). However, no participants in either group exhibited a reduction in SCORAD total score of more than 75% at any time point (Supplementary Table 4). In addition, disease severity and IGA score were also improved in the treatment group compared to the placebo group (Fig. 2e-f).

### Biomarker

**Fig. 3:**
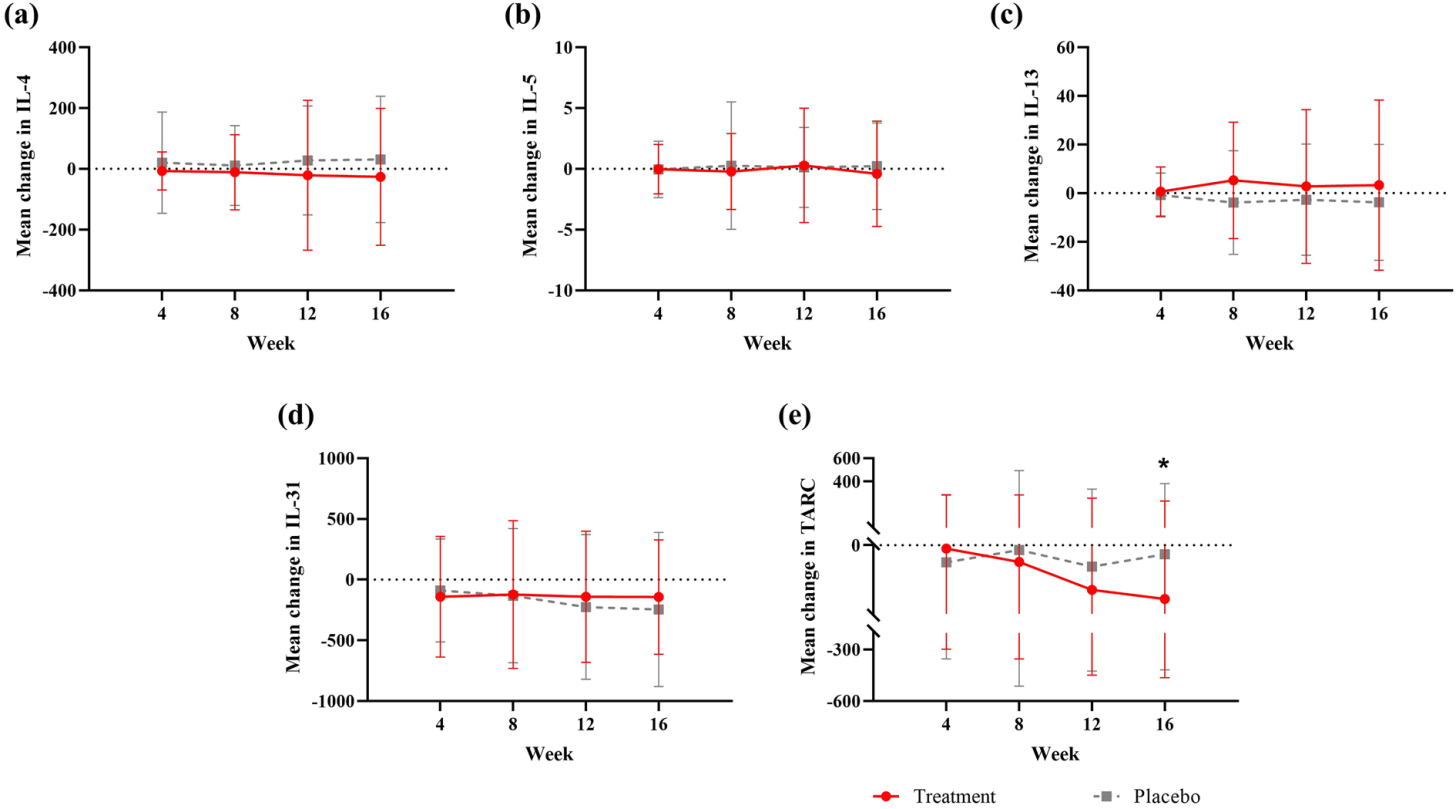
Mean change in blood levels from baseline at each time point: **a** IL-4, **b** IL-5, **c** IL-13, **d** IL-31 and **e** TARC. ^*^P < .05. *P*-values are presented only when differences are statistically significant.* TARC* thymus and activation regulated chemokine

No biomarkers were significantly different between the treatment and placebo groups at baseline. The change in IL-4, 5, 13, 31 levels from baseline to week 4, 8, 12, and 16 was not significantly different at any time point. However, the change from baseline to week 16 in TARC levels was significantly different between the treatment and placebo groups (*P* = .012) (Fig. 3a–e). The changes from baseline to week 4, 8, 12, and 16 in blood PGE2, ECP, TGF-β1, IL-6, 8 levels were not significantly different at any time point (Supplementary Fig. 1a–e).

### Safety assessment

Of the 114 patients included in the SS, 29 (25.4%) experienced 43 treatment-emergent adverse events (TEAEs) and 5 (4.4%) experienced 10 adverse drug reactions (ADRs), with no significant difference in the incidence of TEAEs and ADRs between the treatment and placebo groups (Table 2 and Supplementary Table 5). The most common ADR was ‘headache’ in 3 participants (2.6%), followed by ‘dizziness,’ ‘urticaria,’ ‘vitiligo,’ ‘nausea,’ and ‘phlebitis,’ each in 1 participant (0.9%). Adverse reactions that led to discontinuation of the medication occurred in 1 participant (0.9%, 2 cases) and were not considered ADRs. There were no significant differences between the groups in the incidence of adverse reactions leading to discontinuation of the medication, and no serious adverse events (SAEs) occurred. No significant findings were observed in vital signs, laboratory tests, and physical examinations.

## Discussion

The pathophysiology of AD is multifaceted, involving genetic, epidermal barrier, and immunopathogenic factors [1]. The activation of T helper 2 (Th2) lymphocytes and the cytokines they release contribute to increased production of IgE from B cells, heightened skin inflammation, and worsening of the skin barrier defect in AD, underscoring the pivotal role of lymphocytes in the immunopathogenic abnormalities [1]. AD is characterized by a plethora of cytokines, including IL-4, 13, 17, 22, 31, and thymic stromal lymphopoietin (TSLP) [21, 22]. The production of IL-17, along with cytokines from Th1 and Th2 cells, has been associated with the pathogenesis of Asian AD [23]. Mast cells further participate in the pathogenesis of AD [24].

Currently, various regenerative medicine approaches exist and have shown promise in the treatment of inflammatory skin diseases and scar healing [25–29]. In this study, MSC infusion was evaluated as a particularly promising strategy, and among them, we specifically utilized adipose-derived AtMSCs. MSCs are implicated in both innate and adaptive immunity and exert their immunomodulatory functions primarily through direct interactions with immune cells and paracrine activity [12, 30]. Treatment with superoxide dismutase 3-transduced MSCs has been shown to limit T cell infiltration into the skin and reduce the numbers of CD4^+^ and CD8^+^ T cells in the spleen and lymph node in BALB/c mice with AD induced by ovalbumin [31]. It is evidenced that intravenous infusion of MSCs can forestall the production of IgE by B cells, thus preventing AD [17, 32]. Additionally, MSC-treated NC/Nga mice with AD induced by *Dermatophagoides farina* (Df) exhibited a decline in IgE levels in serum [20, 33]. MSCs have been shown to inhibit mast cell degranulation in animal models of AD [17, 33, 34]. Subcutaneous injections of MSCs led to a reduction in both the total number of degranulated mast cells and their degranulation rate in NC/Nga mice with AD caused by Df [33, 34]. Pre-clinical studies suggest that MSC therapy is a promising approach for AD treatment, demonstrating therapeutic efficacy and elucidating the mechanisms involved.

This represents the first extensive phase 2 investigation, to our knowledge, demonstrating that AtMSCs administered to patients with moderate to severe AD are markedly effective in diminishing EASI, SCORAD, disease severity, and IGA scores compared with a placebo, all without significant complications. The recent phase 1/2 clinical trial using allogeneic human bone marrow-derived clonal mesenchymal stem cells for the treatment of AD reported similar efficacy to the 16-week EASI-75 response rate observed in this study [35]. Compared to bone marrow-derived MSCs, the AtMSCs used in this study are simpler to harvest from the patient’s own tissues, and based on reports indicating that HLA-matched cell therapy elicits less immune response and has higher survival rates than mismatched cell therapy [36], it is expected that the risk of rejection will be reduced, and long-term safety and efficacy will be maintained.

Notably, among blood cytokines, TARC, also known as CCL17 and recognized as pivotal AD clinical biomarkers [37, 38], exhibited substantial differences in the study group relative to the placebo cohort at the 16-week mark following administration of the investigational drug. TARC is a chemokine with potent chemotactic activity for Th2 cells and is well known to be closely associated with disease activity in AD [39]. In particular, TARC has been reported as an important early indicator preceding the onset of AD in pediatric and adolescent patients [40]. AtMSC therapy may have improved AD by reducing TARC levels, thereby suppressing the infiltration of pathogenic Th2 cells into the skin and leading to clinical improvement. However, in this study, no significant changes were observed in the serum levels of other Th2-related cytokines such as IL-4, IL-5, IL-13, and IL-31. One possible explanation is that the reduction in TARC by AtMSC therapy primarily inhibited the infiltration of Th2 cells, and the subsequent changes in cytokine production may require a longer observation period to become evident. Another possibility is that AtMSC therapy had a limited impact on cytokines already being produced by Th2 cells that had infiltrated the skin prior to treatment. Moreover, in the pathophysiology of AD, cytokine concentrations in skin tissue may carry greater significance than those in serum. Therefore, future studies should directly assess cytokine changes at the tissue level. To determine whether the TARC reduction induced by AtMSC therapy ultimately translates into measurable changes in these Th2-related cytokines, long-term follow-up studies such as phase 3 clinical trials will be necessary.

There are currently no studies directly comparing the efficacy of treatment of AtMSC and existing biologics for atopic dermatitis. However, based on results reported in the literature, the proportion achieving EASI-75 at week 16 was 43.3% for dupilumab with corticosteroids [41], 33.2% for tralokinumab (ECZTRA 2) [10], and 48% for the 4 mg baricitinib with corticosteroids [42]. In this study, the AtMSC treatment group showed an EASI-75 response rate of 23.7% at week 16, which was comparable to the results reported for existing biologics and JAK inhibitors. These findings suggest that AtMSC could be a promising alternative or adjunctive treatment option in the clinical setting for AD therapy.

Limitations of this study include its single-blind design, with future phase 3 trials planned to adopt a double-blind design. In addition, IFN-γ and IL-17 levels, which may be influenced by MSCs, were not examined [43–45]. Previous studies have reported that patients with higher IL-17 levels tend to respond more favorably to MSC therapy, suggesting the need for further research to determine whether IL-17 could serve as an effective biomarker for selecting MSC treatment in moderate to severe AD cases, as well as for long-term observational studies on MSC therapy [19]. Moreover, the evaluation period in this study was limited to 16 weeks, making it difficult to assess the long-term durability and relapse rates of AtMSC. To address this limitation, the upcoming phase 3 clinical trial will include extended efficacy assessments and long-term follow-up to further evaluate the sustained therapeutic effects.

In conclusion, AtMSC has the potential to effectively improve moderate to severe AD without significant complications. The immunomodulatory properties of MSCs render them a promising therapeutic strategy, which might be applicable to chronic inflammatory diseases, including AD. Furthermore, infused MSCs are likely to exert long-term effects in vivo, supporting their potential as a durable treatment option for AD.

**Table 2:** Summary of TEAEs

MedDRA System organ class Preferred term	Treatment group (*N* = 59)	Placebo group (*N* = 55)	Total (*N* = 114)
Any TEAEs, n(%)[event]	17(28.8)[26]	12(21.8)[17]	29(25.4)[43]
Infections and infestations	6(10.2)[7]	7(12.7)[7]	13(11.4)[14]
COVID-19	2(3.4)[2]	2(3.6)[2]	4(3.5)[4]
Cellulitis	1(1.7)[1]	0(0.0)[0]	1(0.9)[1]
Eczema herpeticum	0(0.0)[0]	1(1.8)[1]	1(0.9)[1]
Herpes simplex	1(1.7)[1]	0(0.0)[0]	1(0.9)[1]
Herpes zoster	0(0.0)[0]	1(1.8)[1]	1(0.9)[1]
Myringitis	1(1.7)[1]	0(0.0)[0]	1(0.9)[1]
Nasopharyngitis	0(0.0)[0]	1(1.8)[1]	1(0.9)[1]
Otitis externa	1(1.7)[1]	0(0.0)[0]	1(0.9)[1]
Rhinitis	1(1.7)[1]	0(0.0)[0]	1(0.9)[1]
Tinea manuum	0(0.0)[0]	1(1.8)[1]	1(0.9)[1]
Vaginal infection	0(0.0)[0]	1(1.8)[1]	1(0.9)[1]
Skin and subcutaneous tissue disorders	4(6.8)[4]	2(3.6)[2]	6(5.3)[6]
Acne	1(1.7)[1]	0(0.0)[0]	1(0.9)[1]
Dermatitis acneiform	0(0.0)[0]	1(1.8)[1]	1(0.9)[1]
Dermatitis contact	0(0.0)[0]	1(1.8)[1]	1(0.9)[1]
Epidermolysis	1(1.7)[1]	0(0.0)[0]	1(0.9)[1]
Urticaria	1(1.7)[1]	0(0.0)[0]	1(0.9)[1]
Vitiligo	1(1.7)[1]	0(0.0)[0]	1(0.9)[1]
Nervous system disorders	3(5.1)[6]	0(0.0)[0]	3(2.6)[6]
Headache	3(5.1)[4]	0(0.0)[0]	3(2.6)[4]
Dizziness	1(1.7)[2]	0(0.0)[0]	1(0.9)[2]
Investigations	1(1.7)[2]	2(3.6)[3]	3(2.6)[5]
Alanine aminotransferase increased	1(1.7)[1]	2(3.6)[2]	3(2.6)[3]
Aspartate aminotransferase increased	1(1.7)[1]	1(1.8)[1]	2(1.8)[2]
Gastrointestinal disorders	2(3.4)[2]	1(1.8)[1]	3(2.6)[3]
Gastrooesophageal reflux disease	0(0.0)[0]	1(1.8)[1]	1(0.9)[1]
Nausea	1(1.7)[1]	0(0.0)[0]	1(0.9)[1]
Oesophagitis	1(1.7)[1]	0(0.0)[0]	1(0.9)[1]
Vascular disorders	2(3.4)[2]	1(1.8)[1]	3(2.6)[3]
Hypertension	1(1.7)[1]	1(1.8)[1]	2(1.8)[2]
Phlebitis	1(1.7)[1]	0(0.0)[0]	1(0.9)[1]
Eye disorders	1(1.7)[1]	0(0.0)[0]	1(0.9)[1]
Conjunctivitis allergic	1(1.7)[1]	0(0.0)[0]	1(0.9)[1]
Injury, poisoning and procedural complications	1(1.7)[1]	0(0.0)[0]	1(0.9)[1]
Skin abrasion	1(1.7)[1]	0(0.0)[0]	1(0.9)[1]
Metabolism and nutrition disorders	0(0.0)[0]	1(1.8)[1]	1(0.9)[1]
Gout	0(0.0)[0]	1(1.8)[1]	1(0.9)[1]
Musculoskeletal and connective tissue disorders	1(1.7)[1]	0(0.0)[0]	1(0.9)[1]
Muscle spasms	1(1.7)[1]	0(0.0)[0]	1(0.9)[1]
Respiratory, thoracic and mediastinal disorders	0(0.0)[0]	1(1.8)[1]	1(0.9)[1]
Asthma	0(0.0)[0]	1(1.8)[1]	1(0.9)[1]
Life-threatening TEAE	0(0.0)[0]	0(0.0)[0]	0(0.0)[0]
Serious ADR	0(0.0)[0]	0(0.0)[0]	0(0.0)[0]
Severity, n(%)[event]	17(28.8)[26]	^†^12(21.8)[17]	^†^29(25.4)[43]
Mild	16(27.1)[24]	^†^11(20.0)[14]	^†^27(23.7)[38]
Moderate	1(1.7)[2]	^†^2(3.6)[3]	^†^3(2.6)[5]
Severe	0	0	0

Adverse events are coded according to MedDRA 25.1

Percentages are based on the number of subjects in the treatment group

^†^Two adverse events of different severities (mild and moderate) occurred in one participant in the placebo group

## Supplementary Information

Below is the link to the electronic supplementary material.

Supplementary Material 1. Mean change in blood levels from baseline at each time point: (**a**) PGE2, (**b**) ECP, (**c**) TGF-β1, (**d**) IL-6 and (**e**) IL-8. PGE2, Prostaglandin E2; ECP, Eosinophil Cationic Protein; TGF-β1, Tumor growth factor-β1.

Supplementary Material 2

## Data Availability

No datasets were generated or analysed during the current study.

## Abbreviations

AD: Atopic dermatitis
MSC: Mesenchymal stem cells
AtMSC: Autologous adipose tissue-derived MSC
EASI: Eczema Area and Severity Index
SCORAD: Scoring Atopic Dermatitis
IGA: Investigator’s Global Assessment
PGE2: Prostaglandin E2
ECP: Eosinophil Cationic Protein
TEAEs: Treatment-emergent adverse events
ADRs: Adverse drug reactions
SAEs: Serious adverse events
Th2: T helper 2
TSLP: Thymic stromal lymphopoietin
Df: Dermatophagoides farina
TARC (CCL17): Thymus and activation regulated chemokine
