# Supplementary figures and images for: Efficacy and safety of autologous adipose-derived stem cells in subjects with moderate to severe atopic dermatitis: a multicenter, randomized, single-blind, placebo-controlled, phase 2 trial
Source: Stem Cell Res Ther. 2025 Dec 2;16:671. doi: 10.1186/s13287-025-04763-y (PMC12673732; doi:10.1186/s13287-025-04763-y)

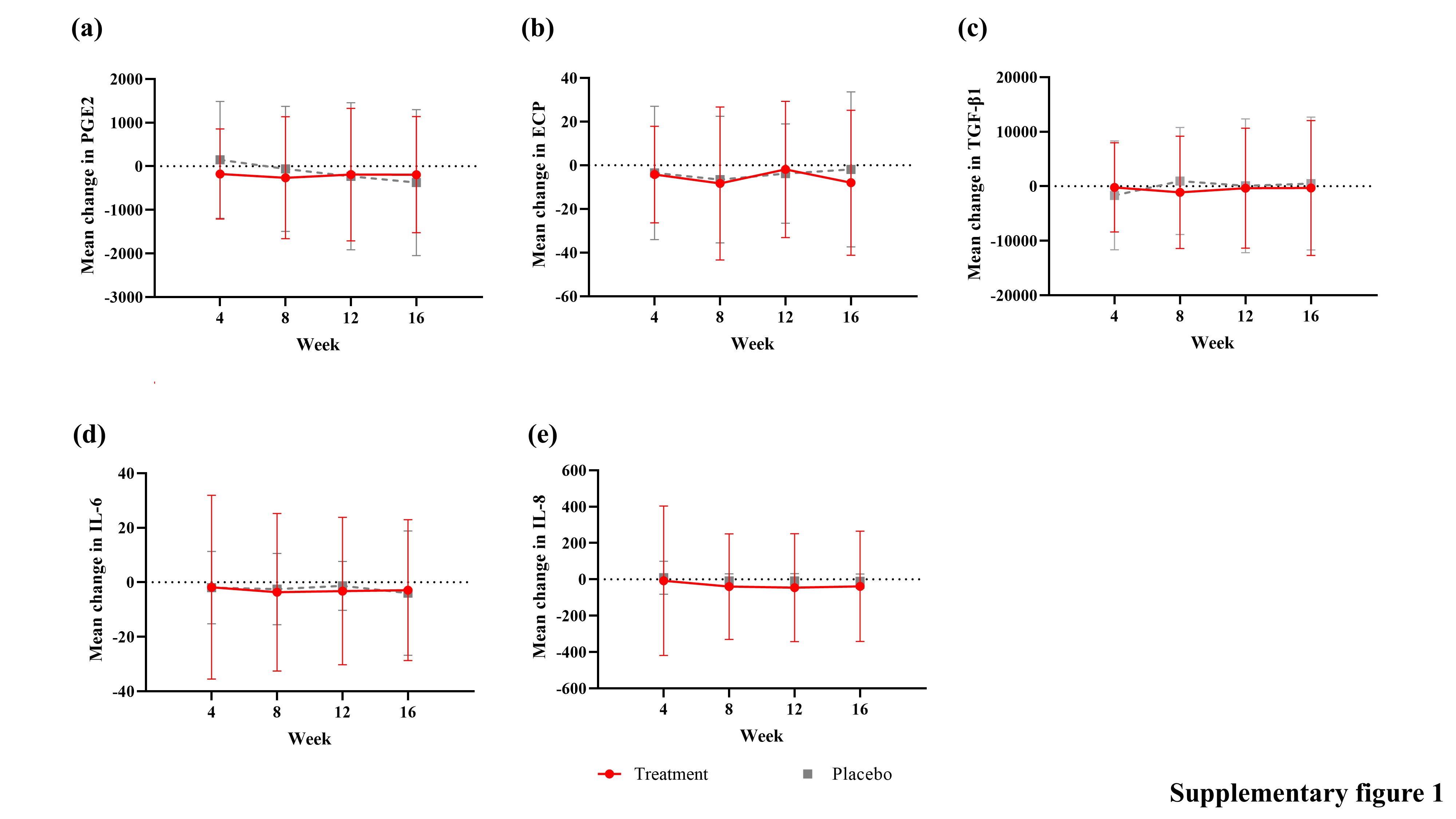

Supplement: Supplementary file 1 — Supplementary Material 1. Mean change in blood levels from baseline at each time point: (a) PGE2, (b) ECP, (c) TGF-β1, (d) IL-6 and (e) IL-8. PGE2, Prostaglandin E2; ECP, Eosinophil Cationic Protein; TGF-β1, Tumor growth factor-β1. [file 13287_2025_4763_MOESM1_ESM.jpg]
