# Supplementary material for: Efficacy and safety of autologous adipose-derived stem cells in subjects with moderate to severe atopic dermatitis: a multicenter, randomized, single-blind, placebo-controlled, phase 2 trial
Source: Stem Cell Res Ther. 2025 Dec 2;16:671. doi: 10.1186/s13287-025-04763-y (PMC12673732; doi:10.1186/s13287-025-04763-y)
Supplement: Supplementary file 2 — Supplementary Material 2 [file 13287_2025_4763_MOESM2_ESM.docx]

**Supplementary table**

**Supplementary table 1. Subject Disposition (All Screened Subjects)**

| **Subject Disposition** | **Test**  **group** | **Placebo group** | **Total** |
| --- | --- | --- | --- |
| Screened |  |  | 130 |
| Screening Failure |  |  | 12 |
|  | | | |
| Reason for Screening Failure |  |  | 12 |
| Inadequate inclusion/exclusion criteria |  |  | 8 |
| Withdrawing participant consent |  |  | 3 |
| Other^†^ |  |  | 1 |
|  | | | |
| Randomized Subjects | 60 | 58 | 118 |
| Treated | 59 | 55 | 114 |
| Not Treated | 1 | 3 | 4 |
|  | | | |
| Status of Study |  |  |  |
| Completed | 54 | 53 | 107 |
| Discontinued | 6 | 5 | 11 |
|  | | | |
| Reason for Discontinuation |  |  |  |
| Withdrawing consent | 1 | 5 | 6 |
| Violation of inclusion/exclusion criteria | 0 | 0 | 0 |
| Having an acute reaction to the study medication, such as a severe allergy or hypersensitivity reaction | 0 | 0 | 0 |
| Participants having a serious adverse event or requesting to discontinue the study due to an adverse event. | 1 | 0 | 1 |
| Having a systemic disease that was not detected by pre-procedure testing. | 0 | 0 | 0 |
| Participants or their representative requesting discontinuation of the study due to unsatisfactory treatment effect during the study. | 0 | 0 | 0 |
| Investigator or participant having significant protocol violation. | 0 | 0 | 0 |
| Participants unable to track | 0 | 0 | 0 |
| When the investigator believes it is beneficial for the participant to stop the study | 0 | 0 | 0 |
| Other^‡^ | 4 | 0 | 4 |

- Screened out due to subject's vascular status unsuitable for IP, as determined by the investigator.
- Dropped out due to the final quality test found it to be inappropritate.

**Supplementary table 2. Validation methods**

**Eczema Area and Severity Index (EASI)**

At each visit from the screening visit (Visit 1) through the end-of-visit (Visit 6), patients will be asked to score their clinical findings on a 4-point scale (0=none, 1=mild, 2=moderate, 3=severe) for the severity of erythema, induration/papules, epidermal desquamation, and lichen planus on the head/neck, upper extremities, trunk, and lower extremities, 3=severe) and multiplied by the lesion area score per body part (0=none, 1=<10%, 2=10 to 29%, 3=30 to 49%, 4=50 to 69%, 5=70 to 89%, 6=90 to 100%) to assess the degree of improvement for each body part. The scores for each body part were multiplied by 0.1 for the head/neck, 0.2 for the upper extremities, 0.3 for the trunk, and 0.4 for the lower extremities, and then added together to calculate the EASI total score.

| **Body region** | **EASI Score** | E = Erythema  I = Induration/Papulation  Ex = Excoriation  L = Lichenification |
| --- | --- | --- |
| Head/Neck (H) | (E+I+Ex+L) x Area x 0.1 |  |
| Upper limbs (UL) | (E+I+Ex+L) x Area x 0.2 |  |
| Trunk (T) | (E+I+Ex+L) x Area x 0.3 |  |
| Lower limbs (LL) | (E+I+Ex+L) x Area x 0.4 |  |
| EASI = | Sum of above 4 body region scores |  |

**Scoring Atopic Dermatitis (SCORAD)**

At each visit, from the screening visit (Visit 1) to the exit visit (Visit 6), we evaluated the following.

| **SCORAD Index = (A/5 + 7B/2 +C)** | |
| --- | --- |
| A: Extent Criteria | Skin involvement was determined by the rule of nine. |
| B: Intensity Criteria (0~18) | The severity of each symptom of atopic dermatitis was rated on a scale of 0 to 3.   \|  \| **None** \| **Mild** \| **Moderate** \| **Severe** \| \| --- \| --- \| --- \| --- \| --- \| \| Erythema \| 0 \| 1 \| 2 \| 3 \| \| Edema/Papulation \| 0 \| 1 \| 2 \| 3 \| \| Oozing/crusting \| 0 \| 1 \| 2 \| 3 \| \| Excoriation \| 0 \| 1 \| 2 \| 3 \| \| Lichenification \| 0 \| 1 \| 2 \| 3 \| \| Dryness \| 0 \| 1 \| 2 \| 3 \| |
| C: Subject symptoms  (For three days and nights prior to the visit) | Subjective symptoms of pruritus and insomnia were rated on a 0-10 scale by the examiner. |

**Grading of the severity of atopic dermatitis**

Each visit, from baseline/dosing visit (Visit 2) to end of treatment visit (Visit 6), the severity of atopic dermatitis was evaluated according to the Rajka and Langeland criteria.

| **Grading of the severity of atopic dermatitis (data from Rajka and Langeland)** | | |
| --- | --- | --- |
|  |  | **Score*** |
| Extent | Involvement is less than 9% of body surface area | 1 |
|  | Involvement is greater than 9% of body surface area, but less than 36%. | 2 |
|  | Involvement is greater than 36% of body surface area | 3 |
| Progress | ≥ 3 months in a year without atopic symptoms | 1 |
|  | < 3 months in a year without atopic symptoms | 2 |
|  | Persistent atopic dermatitis symptoms | 3 |
| Severity | Mild pruritus with intermittent awakening at night | 1 |
|  | Pruritus severity ≥ 1 and < 3 | 2 |
|  | Severe pruritus with often awakening at night | 3 |
| Total score | 3 ~ 4 | Mild |
|  | 4.5 ~ 7.5 | Moderate |
|  | 8 ~ 9 | Severe |
| * 1.5 to 2.5 scores are also available | | |

**Investigator’s Global Assessment (IGA)**

Each visit, from the baseline/dosing visit (Visit 2) to the end of the study (Visit 6), IGA was rated on a scale of 0 (not severe) to 5 (very severe).

| **Score** | | **Description** |
| --- | --- | --- |
| 0 | Clear | No inflammatory signs of atopic dermatitis |
| 1 | Almost clear | Just perceptible erythema, and just perceptible papulation/infiltration |
| 2 | Mild disease | Mild erythema, and mild papulation/infiltration |
| 3 | Moderate disease | Moderate erythema, and moderate papulation/infiltration |
| 4 | Severe disease | Severe erythema, and severe papulation/infiltration |
| 5 | Very severe disease | Severe erythema, and severe papulation/infiltration with oozing/crusting |

**Supplementary table 3. Concurrent Disease (Full Analysis Set)**

| **MedDRA System Organ Class  Preferred Term** | **Treatment**  **group (N=59)** | **Placebo**  **group (N=55)** | **Total (N=114)** |
| --- | --- | --- | --- |
| Subjects with Concurrent Disease, n(%)[event] | 41(69.5)[155] | 35(63.6)[116] | 76(66.7)[271] |
|  | | | |
| Immune system disorders | 23(39.0)[104] | 17(30.9)[65] | 40(35.1)[169] |
| Mite allergy | 20(33.9)[62] | 15(27.3)[42] | 35(30.7)[104] |
| Allergy to animal | 13(22.0)[20] | 7(12.7)[11] | 20(17.5)[31] |
| Food allergy | 9(15.3)[16] | 5(9.1)[5] | 14(12.3)[21] |
| Cockroach allergy | 3(5.1)[3] | 1(1.8)[1] | 4(3.5)[4] |
| Dust allergy | 1(1.7)[1] | 1(1.8)[1] | 2(1.8)[2] |
| Milk allergy | 1(1.7)[1] | 1(1.8)[1] | 2(1.8)[2] |
| Mycotic allergy | 0(0.0)[0] | 2(3.6)[2] | 2(1.8)[2] |
| Allergy to plants | 0(0.0)[0] | 1(1.8)[1] | 1(0.9)[1] |
| Hypersensitivity | 0(0.0)[0] | 1(1.8)[1] | 1(0.9)[1] |
| Seasonal allergy | 1(1.7)[1] | 0(0.0)[0] | 1(0.9)[1] |
|  | | | |
| Respiratory, thoracic and mediastinal disorders | 9(15.2)[10] | 9(16.4)[11] | 18(15.8)[21] |
| Rhinitis allergic | 8(13.6)[8] | 8(14.6)[8] | 16(14.0)[16] |
| Asthma | 1(1.7)[1] | 3(5.5)[3] | 4(3.5)[4] |
| Cough variant asthma | 1(1.7)[1] | 0(0.0)[0] | 1(0.9)[1] |
|  | | | |
| Infections and infestations | 5(8.5)[5] | 5(9.1)[6] | 10(8.8)[11] |
| Rhinitis | 4(6.8)[4] | 3(5.5)[3] | 7(6.1)[7] |
| COVID-19 | 0(0.0)[0] | 2(3.6)[2] | 2(1.8)[2] |
| Hordeolum | 1(1.7)[1] | 0(0.0)[0] | 1(0.9)[1] |
| Tinea cruris | 0(0.0)[0] | 1(1.8)[1] | 1(0.9)[1] |
|  | | | |
| Metabolism and nutrition disorders | 4(6.8)[5] | 6(10.9)[6] | 10(8.8)[11] |
| Gout | 3(5.1)[3] | 0(0.0)[0] | 3(2.6)[3] |
| Hyperlipidaemia | 1(1.7)[1] | 2(3.6)[2] | 3(2.6)[3] |
| Hyperuricaemia | 0(0.0)[0] | 3(5.5)[3] | 3(2.6)[3] |
| Diabetes mellitus | 1(1.7)[1] | 1(1.8)[1] | 2(1.8)[2] |
|  | | | |
| Vascular disorders | 2(3.4)[2] | 6(10.9)[6] | 8(7.0)[8] |
| Hypertension | 2(3.4)[2] | 6(10.9)[6] | 8(7.0)[8] |
|  | | | |
| Skin and subcutaneous tissue disorders | 3(5.1)[6] | 2(3.6)[4] | 5(4.4)[10] |
| Dermatitis acneiform | 1(1.7)[1] | 1(1.8)[1] | 2(1.8)[2] |
| Acne | 0(0.0)[0] | 1(1.8)[1] | 1(0.9)[1] |
| Alopecia | 0(0.0)[0] | 1(1.8)[1] | 1(0.9)[1] |
| Cutaneous amyloidosis | 1(1.7)[1] | 0(0.0)[0] | 1(0.9)[1] |
| Hand dermatitis | 1(1.7)[1] | 0(0.0)[0] | 1(0.9)[1] |
| Hyperhidrosis | 1(1.7)[1] | 0(0.0)[0] | 1(0.9)[1] |
| Nail dystrophy | 1(1.7)[1] | 0(0.0)[0] | 1(0.9)[1] |
| Neurodermatitis | 1(1.7)[1] | 0(0.0)[0] | 1(0.9)[1] |
| Skin striae | 0(0.0)[0] | 1(1.8)[1] | 1(0.9)[1] |
|  | | | |
| Psychiatric disorders | 4(6.8)[4] | 1(1.8)[1] | 5(4.4)[5] |
| Depression | 3(5.1)[3] | 0(0.0)[0] | 3(2.6)[3] |
| Premature ejaculation | 0(0.0)[0] | 1(1.8)[1] | 1(0.9)[1] |
| Tic | 1(1.7)[1] | 0(0.0)[0] | 1(0.9)[1] |
|  | | | |
| Eye disorders | 3(5.1)[4] | 1(1.8)[1] | 4(3.5)[5] |
| Cataract | 1(1.7)[1] | 0(0.0)[0] | 1(0.9)[1] |
| Dacryostenosis acquired | 1(1.7)[1] | 0(0.0)[0] | 1(0.9)[1] |
| Dry eye | 1(1.7)[1] | 0(0.0)[0] | 1(0.9)[1] |
| Glaucoma | 1(1.7)[1] | 0(0.0)[0] | 1(0.9)[1] |
| Retinal disorder | 0(0.0)[0] | 1(1.8)[1] | 1(0.9)[1] |
|  | | | |
| Injury, poisoning and procedural complications | 1(1.7)[1] | 3(5.5)[4] | 4(3.5)[5] |
| Auricular haematoma | 1(1.7)[1] | 0(0.0)[0] | 1(0.9)[1] |
| Extraskeletal ossification | 0(0.0)[0] | 1(1.8)[1] | 1(0.9)[1] |
| Humerus fracture | 0(0.0)[0] | 1(1.8)[1] | 1(0.9)[1] |
| Ligament sprain | 0(0.0)[0] | 1(1.8)[1] | 1(0.9)[1] |
| Muscle rupture | 0(0.0)[0] | 1(1.8)[1] | 1(0.9)[1] |
|  | | | |
| Cardiac disorders | 2(3.4)[2] | 2(3.6)[2] | 4(3.5)[4] |
| Left ventricular hypertrophy | 1(1.7)[1] | 1(1.8)[1] | 2(1.8)[2] |
| Hypertensive heart disease | 1(1.7)[1] | 0(0.0)[0] | 1(0.9)[1] |
| Myocardial infarction | 0(0.0)[0] | 1(1.8)[1] | 1(0.9)[1] |
|  | | | |
| Musculoskeletal and connective tissue disorders | 1(1.7)[1] | 3(5.5)[3] | 4(3.5)[4] |
| Arthralgia | 0(0.0)[0] | 1(1.8)[1] | 1(0.9)[1] |
| Intervertebral disc protrusion | 1(1.7)[1] | 0(0.0)[0] | 1(0.9)[1] |
| Pain in extremity | 0(0.0)[0] | 1(1.8)[1] | 1(0.9)[1] |
| Scoliosis | 0(0.0)[0] | 1(1.8)[1] | 1(0.9)[1] |
|  | | | |
| Reproductive system and breast disorders | 2(3.4)[2] | 2(3.6)[2] | 4(3.5)[4] |
| Amenorrhoea | 0(0.0)[0] | 1(1.8)[1] | 1(0.9)[1] |
| Dysmenorrhoea | 1(1.7)[1] | 0(0.0)[0] | 1(0.9)[1] |
| Erectile dysfunction | 0(0.0)[0] | 1(1.8)[1] | 1(0.9)[1] |
| Polycystic ovaries | 1(1.7)[1] | 0(0.0)[0] | 1(0.9)[1] |
|  | | | |
| Gastrointestinal disorders | 2(3.4)[2] | 1(1.8)[1] | 3(2.6)[3] |
| Chronic gastritis | 1(1.7)[1] | 1(1.8)[1] | 2(1.8)[2] |
| Gastritis | 1(1.7)[1] | 0(0.0)[0] | 1(0.9)[1] |
|  | | | |
| Neoplasms benign, malignant and unspecified (incl cysts and polyps) | 1(1.7)[1] | 2(3.6)[2] | 3(2.6)[3] |
| Benign neoplasm of thyroid gland | 0(0.0)[0] | 1(1.8)[1] | 1(0.9)[1] |
| Neurofibroma | 0(0.0)[0] | 1(1.8)[1] | 1(0.9)[1] |
| Skin papilloma | 1(1.7)[1] | 0(0.0)[0] | 1(0.9)[1] |
|  | | | |
| Endocrine disorders | 2(3.4)[3] | 0(0.0)[0] | 2(1.8)[3] |
| Hyperthyroidism | 1(1.7)[1] | 0(0.0)[0] | 1(0.9)[1] |
| Hypothyroidism | 1(1.7)[1] | 0(0.0)[0] | 1(0.9)[1] |
| Thyroid mass | 1(1.7)[1] | 0(0.0)[0] | 1(0.9)[1] |
|  | | | |
| Investigations | 1(1.7)[1] | 1(1.8)[1] | 2(1.8)[2] |
| Hepatitis B core antibody positive | 0(0.0)[0] | 1(1.8)[1] | 1(0.9)[1] |
| Hepatitis B virus test positive | 1(1.7)[1] | 0(0.0)[0] | 1(0.9)[1] |
|  | | | |
| Nervous system disorders | 2(3.4)[2] | 0(0.0)[0] | 2(1.8)[2] |
| Hypoaesthesia | 1(1.7)[1] | 0(0.0)[0] | 1(0.9)[1] |
| Myelopathy | 1(1.7)[1] | 0(0.0)[0] | 1(0.9)[1] |
|  | | | |
| General disorders and administration site conditions | 0(0.0)[0] | 1(1.8)[1] | 1(0.9)[1] |
| Chest pain | 0(0.0)[0] | 1(1.8)[1] | 1(0.9)[1] |
|  | | | |
| Concurrent Disease events are coded using MedDRA Version 25.1. | | | |
| Concurrent Disease is displayed as number of subjects, percentage of subjects, and number of events. | | | |
| Percentages are based on the number of subjects in the treatment group. | | | |

**Supplementary table 4. Change from baseline (Visit 2) in SCORAD score by assessment Item (extent criteria, erythema, edema/papulation, oozing/crusting, excoriation, lichenification, dryness, pruritus, insomnia) at weeks 4, 8, 12, and 16 (Full Analysis Set)**

|  | **Treatment**  **group (N=59)** | **Placebo**  **group (N=55)** |
| --- | --- | --- |
| **[ Extent Criteria ]** | | |
| **Baseline** | | |
| n | 59 | 55 |
| Mean(SD) | 33.11(14.59) | 36.24(16.84) |
| Median | 30.00 | 35.00 |
| Min, Max | 9.00, 71.00 | 9.00, 84.00 |
| P-value [2] | .28[d] |  |
|  | | |
| **Week 4** | | |
| n | 58 | 53 |
| Mean(SD) | 27.98(14.48) | 34.99(16.56) |
| Median | 25.50 | 34.00 |
| Min, Max | 3.00, 74.00 | 4.00, 84.00 |
| P-value [2] | .015[d] |  |
|  | | |
| **Week 8** | | |
| n | 59 | 55 |
| Mean(SD) | 25.88(15.59) | 34.11(17.44) |
| Median | 20.00 | 35.00 |
| Min, Max | 3.00, 73.00 | 3.00, 85.00 |
| P-value [2] | .006[d] |  |
|  | | |
| **Week 12** | | |
| n | 59 | 55 |
| Mean(SD) | 23.93(15.86) | 31.84(18.69) |
| Median | 20.00 | 30.00 |
| Min, Max | 2.00, 72.00 | 2.00, 85.00 |
| P-value [2] | .02[d] |  |
|  | | |
| **Week 16** | | |
| n | 59 | 55 |
| Mean(SD) | 20.27(14.07) | 30.88(18.36) |
| Median | 17.00 | 30.00 |
| Min, Max | 3.00, 81.00 | 1.00, 85.00 |
| P-value [2] | <.001[d] |  |
|  | | |
| **Change from Baseline at Week 4** | | |
| n | 58 | 53 |
| Mean(SD) | -5.37(10.84) | -1.59(9.93) |
| Median | -4.00 | -2.00 |
| Min, Max | -52.00, 20.00 | -22.00, 39.00 |
| P-value [1] | <.001[b] | .034[b] |
| P-value [2] | .15[d] |  |
|  | | |
| **Change from Baseline at Week 8** | | |
| n | 59 | 55 |
| Mean(SD) | -7.23(14.16) | -2.12(11.69) |
| Median | -6.00 | -2.00 |
| Min, Max | -64.00, 30.00 | -31.00, 39.00 |
| P-value [1] | <.001[b] | .054[b] |
| P-value [2] | .047[d] |  |
|  | | |
| **Change from Baseline at Week 12** | | |
| n | 59 | 55 |
| Mean(SD) | -9.18(12.63) | -4.39(13.86) |
| Median | -8.00 | -4.00 |
| Min, Max | -48.00, 18.00 | -43.00, 39.00 |
| P-value [1] | <.001[a] | .001[b] |
| P-value [2] | .13[d] |  |
|  | | |
| **Change from Baseline at Week 16** | | |
| n | 59 | 55 |
| Mean(SD) | -12.84(13.09) | -5.36(14.13) |
| Median | -13.00 | -4.00 |
| Min, Max | -45.00, 20.00 | -45.00, 33.00 |
| P-value [1] | <.001[a] | .007[a] |
| P-value [2] | .004[c] |  |
|  | | |
| **[ Erythema ]** | | |
| **Baseline** | | |
| n | 59 | 55 |
| Mean(SD) | 2.00(0.62) | 2.00(0.54) |
| Median | 2.00 | 2.00 |
| Min, Max | 1.00, 3.00 | 1.00, 3.00 |
| P-value [2] | 1.00[d] |  |
|  | | |
| **Week 4** | | |
| n | 58 | 53 |
| Mean(SD) | 1.86(0.58) | 2.04(0.55) |
| Median | 2.00 | 2.00 |
| Min, Max | 1.00, 3.00 | 1.00, 3.00 |
| P-value [2] | .10[d] |  |
|  | | |
| **Week 8** | | |
| n | 59 | 55 |
| Mean(SD) | 1.76(0.68) | 2.05(0.65) |
| Median | 2.00 | 2.00 |
| Min, Max | 1.00, 3.00 | 1.00, 3.00 |
| P-value [2] | .02[d] |  |
|  | | |
| **Week 12** | | |
| n | 59 | 55 |
| Mean(SD) | 1.64(0.64) | 2.04(0.64) |
| Median | 2.00 | 2.00 |
| Min, Max | 1.00, 3.00 | 1.00, 3.00 |
| P-value [2] | .002[d] |  |
|  | | |
| **Week 16** | | |
| n | 59 | 55 |
| Mean(SD) | 1.53(0.70) | 2.00(0.69) |
| Median | 1.00 | 2.00 |
| Min, Max | 0.00, 3.00 | 1.00, 3.00 |
| P-value [2] | <.001[d] |  |
|  | | |
| **Change from Baseline at Week 4** | | |
| n | 58 | 53 |
| Mean(SD) | -0.14(0.54) | 0.06(0.50) |
| Median | 0.00 | 0.00 |
| Min, Max | -1.00, 1.00 | -1.00, 1.00 |
| P-value [1] | .10[b] | .58[b] |
| P-value [2] | .052[d] |  |
|  | | |
| **Change from Baseline at Week 8** | | |
| n | 59 | 55 |
| Mean(SD) | -0.24(0.70) | 0.05(0.65) |
| Median | 0.00 | 0.00 |
| Min, Max | -2.00, 1.00 | -1.00, 2.00 |
| P-value [1] | .010[b] | .68[b] |
| P-value [2] | .04[d] |  |
|  | | |
| **Change from Baseline at Week 12** | | |
| n | 59 | 55 |
| Mean(SD) | -0.36(0.61) | 0.04(0.67) |
| Median | 0.00 | 0.00 |
| Min, Max | -2.00, 1.00 | -1.00, 2.00 |
| P-value [1] | <.001[b] | .69[b] |
| P-value [2] | .002[d] |  |
|  | | |
| **Change from Baseline at Week 16** | | |
| n | 59 | 55 |
| Mean(SD) | -0.47(0.73) | 0.00(0.67) |
| Median | 0.00 | 0.00 |
| Min, Max | -2.00, 1.00 | -1.00, 2.00 |
| P-value [1] | <.001[b] | 1.00[b] |
| P-value [2] | <.001[d] |  |
|  | | |
| **[ Edema/ papulation ]** | | |
| **Baseline** | | |
| n | 59 | 55 |
| Mean(SD) | 1.68(0.54) | 1.60(0.63) |
| Median | 2.00 | 2.00 |
| Min, Max | 1.00, 3.00 | 0.00, 3.00 |
| P-value [2] | .46[d] |  |
|  | | |
| **Week 4** | | |
| n | 58 | 53 |
| Mean(SD) | 1.48(0.54) | 1.57(0.67) |
| Median | 1.50 | 2.00 |
| Min, Max | 0.00, 2.00 | 0.00, 3.00 |
| P-value [2] | .64[d] |  |
|  | | |
| **Week 8** | | |
| n | 59 | 55 |
| Mean(SD) | 1.44(0.70) | 1.60(0.68) |
| Median | 1.00 | 2.00 |
| Min, Max | 0.00, 3.00 | 0.00, 3.00 |
| P-value [2] | .28[d] |  |
|  | | |
| **Week 12** | | |
| n | 59 | 55 |
| Mean(SD) | 1.36(0.71) | 1.58(0.71) |
| Median | 1.00 | 2.00 |
| Min, Max | 0.00, 3.00 | 0.00, 3.00 |
| P-value [2] | .10[d] |  |
|  | | |
| **Week 16** | | |
| n | 59 | 55 |
| Mean(SD) | 1.15(0.69) | 1.49(0.66) |
| Median | 1.00 | 1.00 |
| Min, Max | 0.00, 3.00 | 0.00, 3.00 |
| P-value [2] | .011[d] |  |
|  | | |
| **Change from Baseline at Week 4** | | |
| n | 58 | 53 |
| Mean(SD) | -0.19(0.54) | -0.04(0.52) |
| Median | 0.00 | 0.00 |
| Min, Max | -2.00, 1.00 | -1.00, 2.00 |
| P-value [1] | .02[b] | .79[b] |
| P-value [2] | .19[d] |  |
|  | | |
| **Change from Baseline at Week 8** | | |
| n | 59 | 55 |
| Mean(SD) | -0.24(0.68) | 0.00(0.58) |
| Median | 0.00 | 0.00 |
| Min, Max | -2.00, 1.00 | -1.00, 2.00 |
| P-value [1] | .007[b] | 1.00[b] |
| P-value [2] | .08[d] |  |
|  | | |
| **Change from Baseline at Week 12** | | |
| n | 59 | 55 |
| Mean(SD) | -0.32(0.65) | -0.02(0.71) |
| Median | 0.00 | 0.00 |
| Min, Max | -2.00, 1.00 | -2.00, 2.00 |
| P-value [1] | <.001[b] | .85[b] |
| P-value [2] | .017[d] |  |
|  | | |
| **Change from Baseline at Week 16** | | |
| n | 59 | 55 |
| Mean(SD) | -0.53(0.60) | -0.11(0.74) |
| Median | -1.00 | 0.00 |
| Min, Max | -2.00, 1.00 | -2.00, 1.00 |
| P-value [1] | <.001[b] | .28[b] |
| P-value [2] | .002[d] |  |
|  | | |
| **[ Oozing/ crusting ]** | | |
| **Baseline** | | |
| n | 59 | 55 |
| Mean(SD) | 1.44(0.70) | 1.38(0.68) |
| Median | 1.00 | 1.00 |
| Min, Max | 0.00, 3.00 | 0.00, 3.00 |
| P-value [2] | .56[d] |  |
|  | | |
| **Week 4** | | |
| n | 58 | 53 |
| Mean(SD) | 1.19(0.74) | 1.32(0.70) |
| Median | 1.00 | 1.00 |
| Min, Max | 0.00, 3.00 | 0.00, 3.00 |
| P-value [2] | .40[d] |  |
|  | | |
| **Week 8** | | |
| n | 59 | 55 |
| Mean(SD) | 1.10(0.69) | 1.38(0.76) |
| Median | 1.00 | 1.00 |
| Min, Max | 0.00, 3.00 | 0.00, 3.00 |
| P-value [2] | .03[d] |  |
|  | | |
| **Week 12** | | |
| n | 59 | 55 |
| Mean(SD) | 1.00(0.74) | 1.29(0.76) |
| Median | 1.00 | 1.00 |
| Min, Max | 0.00, 3.00 | 0.00, 3.00 |
| P-value [2] | .03[d] |  |
|  | | |
| **Week 16** | | |
| n | 59 | 55 |
| Mean(SD) | 0.88(0.74) | 1.40(0.74) |
| Median | 1.00 | 1.00 |
| Min, Max | 0.00, 3.00 | 0.00, 3.00 |
| P-value [2] | <.001[d] |  |
|  | | |
| **Change from Baseline at Week 4** | | |
| n | 58 | 53 |
| Mean(SD) | -0.24(0.57) | -0.06(0.60) |
| Median | 0.00 | 0.00 |
| Min, Max | -1.00, 1.00 | -1.00, 1.00 |
| P-value [1] | .001[b] | 0.65[b] |
| P-value [2] | .11[d] |  |
|  | | |
| **Change from Baseline at Week 8** | | |
| n | 59 | 55 |
| Mean(SD) | -0.34(0.73) | 0.00(0.58) |
| Median | 0.00 | 0.00 |
| Min, Max | -2.00, 1.00 | -1.00, 1.00 |
| P-value [1] | <.001[b] | 1.00[b] |
| P-value [2] | .011[d] |  |
|  | | |
| **Change from Baseline at Week 12** | | |
| n | 59 | 55 |
| Mean(SD) | -0.44(0.75) | -0.09(0.78) |
| Median | 0.00 | 0.00 |
| Min, Max | -2.00, 1.00 | -2.00, 2.00 |
| P-value [1] | <.001[b] | .40[b] |
| P-value [2] | .02[d] |  |
|  | | |
| **Change from Baseline at Week 16** | | |
| n | 59 | 55 |
| Mean(SD) | -0.56(0.77) | 0.02(0.73) |
| Median | 0.00 | 0.00 |
| Min, Max | -2.00, 1.00 | -1.00, 2.00 |
| P-value [1] | <.001[b] | .86[b] |
| P-value [2] | <.001[d] |  |
|  | | |
| **[ Excoriation ]** | | |
| **Baseline** | | |
| n | 59 | 55 |
| Mean(SD) | 1.61(0.59) | 1.73(0.59) |
| Median | 2.00 | 2.00 |
| Min, Max | 0.00, 3.00 | 1.00, 3.00 |
| P-value [2] | .36[d] |  |
|  | | |
| **Week 4** | | |
| n | 58 | 53 |
| Mean(SD) | 1.36(0.64) | 1.45(0.67) |
| Median | 1.00 | 1.00 |
| Min, Max | 0.00, 3.00 | 0.00, 3.00 |
| P-value [2] | .40[d] |  |
|  | | |
| **Week 8** | | |
| n | 59 | 55 |
| Mean(SD) | 1.29(0.62) | 1.55(0.72) |
| Median | 1.00 | 2.00 |
| Min, Max | 0.00, 3.00 | 0.00, 3.00 |
| P-value [2] | .03[d] |  |
|  | | |
| **Week 12** | | |
| n | 59 | 55 |
| Mean(SD) | 1.20(0.58) | 1.45(0.69) |
| Median | 1.00 | 1.00 |
| Min, Max | 0.00, 3.00 | 0.00, 3.00 |
| P-value [2] | .03[d] |  |
|  | | |
| **Week 16** | | |
| n | 59 | 55 |
| Mean(SD) | 1.14(0.60) | 1.49(0.57) |
| Median | 1.00 | 1.00 |
| Min, Max | 0.00, 2.00 | 0.00, 3.00 |
| P-value [2] | .003[d] |  |
|  | | |
| **Change from Baseline at Week 4** | | |
| n | 58 | 53 |
| Mean(SD) | -0.24(0.60) | -0.28(0.60) |
| Median | 0.00 | 0.00 |
| Min, Max | -1.00, 1.00 | -2.00, 1.00 |
| P-value [1] | .002[b] | .002[b] |
| P-value [2] | .84[d] |  |
|  | | |
| **Change from Baseline at Week 8** | | |
| n | 59 | 55 |
| Mean(SD) | -0.32(0.71) | -0.18(0.64) |
| Median | 0.00 | 0.00 |
| Min, Max | -2.00, 1.00 | -2.00, 1.00 |
| P-value [1] | <.001[b] | .04[b] |
| P-value [2] | .20[d] |  |
|  | | |
| **Change from Baseline at Week 12** | | |
| n | 59 | 55 |
| Mean(SD) | -0.41(0.70) | -0.27(0.68) |
| Median | 0.00 | 0.00 |
| Min, Max | -2.00, 1.00 | -2.00, 1.00 |
| P-value [1] | <.001[b] | .003[b] |
| P-value [2] | .30[d] |  |
|  | | |
| **Change from Baseline at Week 16** | | |
| n | 59 | 55 |
| Mean(SD) | -0.47(0.70) | -0.24(0.61) |
| Median | -1.00 | 0.00 |
| Min, Max | -2.00, 1.00 | -1.00, 1.00 |
| P-value [1] | <.001[b] | .004[b] |
| P-value [2] | .047[d] |  |
|  | | |
| **[ Lichenification ]** | | |
| **Baseline** | | |
| n | 59 | 55 |
| Mean(SD) | 2.08(0.57) | 2.02(0.59) |
| Median | 2.00 | 2.00 |
| Min, Max | 1.00, 3.00 | 1.00, 3.00 |
| P-value [2] | .55[d] |  |
|  | | |
| **Week 4** | | |
| n | 58 | 53 |
| Mean(SD) | 1.84(0.52) | 1.92(0.62) |
| Median | 2.00 | 2.00 |
| Min, Max | 1.00, 3.00 | 1.00, 3.00 |
| P-value [2] | .50[d] |  |
|  | | |
| **Week 8** | | |
| n | 59 | 55 |
| Mean(SD) | 1.80(0.58) | 1.76(0.67) |
| Median | 2.00 | 2.00 |
| Min, Max | 1.00, 3.00 | 0.00, 3.00 |
| P-value [2] | .80[d] |  |
|  | | |
| **Week 12** | | |
| n | 59 | 55 |
| Mean(SD) | 1.68(0.63) | 1.80(0.65) |
| Median | 2.00 | 2.00 |
| Min, Max | 0.00, 3.00 | 1.00, 3.00 |
| P-value [2] | .38[d] |  |
|  | | |
| **Week 16** | | |
| n | 59 | 55 |
| Mean(SD) | 1.56(0.68) | 1.78(0.69) |
| Median | 1.00 | 2.00 |
| Min, Max | 0.00, 3.00 | 0.00, 3.00 |
| P-value [2] | .06[d] |  |
|  | | |
| **Change from Baseline at Week 4** | | |
| n | 58 | 53 |
| Mean(SD) | -0.24(0.51) | -0.11(0.51) |
| Median | 0.00 | 0.00 |
| Min, Max | -1.00, 1.00 | -1.00, 1.00 |
| P-value [1] | .001[b] | .18[b] |
| P-value [2] | .19[d] |  |
|  | | |
| **Change from Baseline at Week 8** | | |
| n | 59 | 55 |
| Mean(SD) | -0.29(0.64) | -0.25(0.48) |
| Median | 0.00 | 0.00 |
| Min, Max | -2.00, 1.00 | -1.00, 1.00 |
| P-value [1] | <.001[b] | <.001[b] |
| P-value [2] | .81[d] |  |
|  | | |
| **Change from Baseline at Week 12** | | |
| n | 59 | 55 |
| Mean(SD) | -0.41(0.65) | -0.22(0.53) |
| Median | 0.00 | 0.00 |
| Min, Max | -3.00, 1.00 | -1.00, 1.00 |
| P-value [1] | <.001[b] | .008[b] |
| P-value [2] | .15[d] |  |
|  | | |
| **Change from Baseline at Week 16** | | |
| n | 59 | 55 |
| Mean(SD) | -0.53(0.60) | -0.24(0.61) |
| Median | -1.00 | 0.00 |
| Min, Max | -2.00, 1.00 | -2.00, 1.00 |
| P-value [1] | <.001[b] | .010[b] |
| P-value [2] | .01[d] |  |
|  | | |
| **[ Dryness ]** | | |
| **Baseline** | | |
| n | 59 | 55 |
| Mean(SD) | 2.00(0.62) | 2.07(0.60) |
| Median | 2.00 | 2.00 |
| Min, Max | 0.00, 3.00 | 1.00, 3.00 |
| P-value [2] | .59[d] |  |
|  | | |
| **Week 4** | | |
| n | 58 | 53 |
| Mean(SD) | 1.86(0.63) | 2.04(0.62) |
| Median | 2.00 | 2.00 |
| Min, Max | 0.00, 3.00 | 1.00, 3.00 |
| P-value [2] | .16[d] |  |
|  | | |
| **Week 8** | | |
| n | 59 | 55 |
| Mean(SD) | 1.75(0.63) | 1.89(0.63) |
| Median | 2.00 | 2.00 |
| Min, Max | 0.00, 3.00 | 0.00, 3.00 |
| P-value [2] | .23[d] |  |
|  | | |
| **Week 12** | | |
| n | 59 | 55 |
| Mean(SD) | 1.73(0.64) | 1.89(0.69) |
| Median | 2.00 | 2.00 |
| Min, Max | 1.00, 3.00 | 0.00, 3.00 |
| P-value [2] | .16[d] |  |
|  | | |
| **Week 16** | | |
| n | 59 | 55 |
| Mean(SD) | 1.53(0.68) | 1.89(0.69) |
| Median | 1.00 | 2.00 |
| Min, Max | 0.00, 3.00 | 0.00, 3.00 |
| P-value [2] | .004[d] |  |
|  | | |
| **Change from Baseline at Week 4** | | |
| n | 58 | 53 |
| Mean(SD) | -0.14(0.66) | -0.06(0.50) |
| Median | 0.00 | 0.00 |
| Min, Max | -2.00, 1.00 | -1.00, 1.00 |
| P-value [1] | .12[b] | .58[b] |
| P-value [2] | .49[d] |  |
|  | | |
| **Change from Baseline at Week 8** | | |
| n | 59 | 55 |
| Mean(SD) | -0.25(0.71) | -0.18(0.43) |
| Median | 0.00 | 0.00 |
| Min, Max | -2.00, 1.00 | -1.00, 1.00 |
| P-value [1] | .006[b] | .006[b] |
| P-value [2] | .73[d] |  |
|  | | |
| **Change from Baseline at Week 12** | | |
| n | 59 | 55 |
| Mean(SD) | -0.27(0.76) | -0.18(0.55) |
| Median | 0.00 | 0.00 |
| Min, Max | -2.00, 1.00 | -1.00, 1.00 |
| P-value [1] | .007[b] | .03[b] |
| P-value [2] | .53[d] |  |
|  | | |
| **Change from Baseline at Week 16** | | |
| n | 59 | 55 |
| Mean(SD) | -0.47(0.80) | -0.18(0.58) |
| Median | 0.00 | 0.00 |
| Min, Max | -2.00, 1.00 | -2.00, 1.00 |
| P-value [1] | <.001[b] | .04[b] |
| P-value [2] | .03[d] |  |
|  | | |
| **[ Pruritus ]** | | |
| **Baseline** | | |
| n | 59 | 55 |
| Mean(SD) | 6.54(1.90) | 7.02(2.06) |
| Median | 7.00 | 7.00 |
| Min, Max | 2.00, 10.00 | 2.00, 10.00 |
| P-value [2] | .09[d] |  |
|  | | |
| **Week 4** | | |
| n | 58 | 53 |
| Mean(SD) | 6.00(2.37) | 6.36(2.30) |
| Median | 6.00 | 7.00 |
| Min, Max | 1.00, 10.00 | 2.00, 10.00 |
| P-value [2] | .28[d] |  |
|  | | |
| **Week 8** | | |
| n | 59 | 55 |
| Mean(SD) | 5.54(2.66) | 6.20(2.41) |
| Median | 6.00 | 6.00 |
| Min, Max | 0.00, 10.00 | 0.00, 10.00 |
| P-value [2] | .14[d] |  |
|  | | |
| **Week 12** | | |
| n | 59 | 55 |
| Mean(SD) | 5.03(2.39) | 6.13(2.55) |
| Median | 5.00 | 7.00 |
| Min, Max | 0.00, 10.00 | 0.00, 10.00 |
| P-value [2] | .01[d] |  |
|  | | |
| **Week 16** | | |
| n | 59 | 55 |
| Mean(SD) | 4.76(2.47) | 6.15(2.38) |
| Median | 5.00 | 6.00 |
| Min, Max | 0.00, 10.00 | 0.00, 10.00 |
| P-value [2] | .002[d] |  |
|  | | |
| **Change from Baseline at Week 4** | | |
| n | 58 | 53 |
| Mean(SD) | -0.57(1.94) | -0.74(1.46) |
| Median | -1.00 | 0.00 |
| Min, Max | -4.00, 4.00 | -5.00, 2.00 |
| P-value [1] | .03[a] | <.001[b] |
| P-value [2] | .93[d] |  |
|  | | |
| **Change from Baseline at Week 8** | | |
| n | 59 | 55 |
| Mean(SD) | -1.00(2.41) | -0.82(1.78) |
| Median | -1.00 | -1.00 |
| Min, Max | -6.00, 4.00 | -5.00, 5.00 |
| P-value [1] | .002[a] | <.001[b] |
| P-value [2] | .53[d] |  |
|  | | |
| **Change from Baseline at Week 12** | | |
| n | 59 | 55 |
| Mean(SD) | -1.51(2.28) | -0.89(1.89) |
| Median | -1.00 | -1.00 |
| Min, Max | -7.00, 3.00 | -5.00, 3.00 |
| P-value [1] | <.001[a] | .001[b] |
| P-value [2] | .11[d] |  |
|  | | |
| **Change from Baseline at Week 16** | | |
| n | 59 | 55 |
| Mean(SD) | -1.78(2.49) | -0.87(2.13) |
| Median | -2.00 | -1.00 |
| Min, Max | -8.00, 4.00 | -7.00, 5.00 |
| P-value [1] | <.001[a] | .004[a] |
| P-value [2] | .04[c] |  |
|  | | |
| **[ Insomnia ]** | | |
| **Baseline** | | |
| n | 59 | 55 |
| Mean(SD) | 5.64(2.48) | 6.29(2.34) |
| Median | 6.00 | 7.00 |
| Min, Max | 0.00, 10.00 | 0.00, 10.00 |
| P-value [2] | .19[d] |  |
|  | | |
| **Week 4** | | |
| n | 58 | 53 |
| Mean(SD) | 5.09(2.99) | 5.87(2.59) |
| Median | 5.00 | 6.00 |
| Min, Max | 0.00, 10.00 | 1.00, 10.00 |
| P-value [2] | .12[d] |  |
|  | | |
| **Week 8** | | |
| n | 59 | 55 |
| Mean(SD) | 4.71(3.01) | 5.58(3.01) |
| Median | 5.00 | 6.00 |
| Min, Max | 0.00, 10.00 | 0.00, 10.00 |
| P-value [2] | .08[d] |  |
|  | | |
| **Week 12** | | |
| n | 59 | 55 |
| Mean(SD) | 4.22(2.60) | 5.53(2.96) |
| Median | 4.00 | 6.00 |
| Min, Max | 0.00, 10.00 | 0.00, 10.00 |
| P-value [2] | .01[d] |  |
|  | | |
| **Week 16** | | |
| n | 59 | 55 |
| Mean(SD) | 3.83(2.76) | 5.58(2.78) |
| Median | 3.00 | 6.00 |
| Min, Max | 0.00, 10.00 | 0.00, 10.00 |
| P-value [2] | .001[d] |  |
|  | | |
| **Change from Baseline at Week 4** | | |
| n | 58 | 53 |
| Mean(SD) | -0.57(2.55) | -0.51(2.28) |
| Median | 0.00 | 0.00 |
| Min, Max | -7.00, 8.00 | -6.00, 7.00 |
| P-value [1] | .07[b] | .08[b] |
| P-value [2] | .97[d] |  |
|  | | |
| **Change from Baseline at Week 8** | | |
| n | 59 | 55 |
| Mean(SD) | -0.93(2.75) | -0.71(2.53) |
| Median | -1.00 | -1.00 |
| Min, Max | -8.00, 5.00 | -9.00, 6.00 |
| P-value [1] | .012[a] | .045[b] |
| P-value [2] | .66[d] |  |
|  | | |
| **Change from Baseline at Week 12** | | |
| n | 59 | 55 |
| Mean(SD) | -1.42(2.34) | -0.76(2.77) |
| Median | -1.00 | -1.00 |
| Min, Max | -9.00, 3.00 | -10.00, 7.00 |
| P-value [1] | <.001[a] | .046[a] |
| P-value [2] | .17[c] |  |
|  | | |
| **Change from Baseline at Week 16** | | |
| n | 59 | 55 |
| Mean(SD) | -1.81(2.75) | -0.71(2.48) |
| Median | -2.00 | -1.00 |
| Min, Max | -10.00, 4.00 | -10.00, 7.00 |
| P-value [1] | <.001[b] | .02[b] |
| P-value [2] | .03[d] |  |
| SD = Standard Deviation, Min = Minimum, Max = Maximum [1] P-value for comparisons between Baseline and post-baseline in each group: [a] Paired T-Test [b] Wilcoxon signed rank test. [2] P-value for comparisons between Placebo group and Treatment group: [c] Two-sample t-test [d] Wilcoxon’s rank sum test. | | |

**Supplementary table 5. ADRs according to SOC and PT (Safety Set)**

| **MedDRA System Organ Class  Preferred Term** | **Treatment**  **group (N=59)** | **Placebo**  **group (N=55)** | **Total (N=114)** |
| --- | --- | --- | --- |
| Subjects with ADRs, n(%)[event] | 5(8.5)[10] | 0(0.0)[0] | 5(4.4)[10] |
|  | | | |
| Nervous system disorders | 3(5.1)[6] | 0(0.0)[0] | 3(2.6)[6] |
| Headache | 3(5.1)[4] | 0(0.0)[0] | 3(2.6)[4] |
| Dizziness | 1(1.7)[2] | 0(0.0)[0] | 1(0.9)[2] |
|  | | | |
| Skin and subcutaneous tissue disorders | 2(3.4)[2] | 0(0.0)[0] | 2(1.8)[2] |
| Urticaria | 1(1.7)[1] | 0(0.0)[0] | 1(0.9)[1] |
| Vitiligo | 1(1.7)[1] | 0(0.0)[0] | 1(0.9)[1] |
|  | | | |
| Gastrointestinal disorders | 1(1.7)[1] | 0(0.0)[0] | 1(0.9)[1] |
| Nausea | 1(1.7)[1] | 0(0.0)[0] | 1(0.9)[1] |
|  | | | |
| Vascular disorders | 1(1.7)[1] | 0(0.0)[0] | 1(0.9)[1] |
| Phlebitis | 1(1.7)[1] | 0(0.0)[0] | 1(0.9)[1] |
| Adverse events are coded according to MedDRA 25.1. | | | |
| Percentages are based on the number of subjects in the treatment group. | | | |
